# The Detection of Bile Acids in the Lungs of Paediatric Cystic Fibrosis Patients Is Associated with Altered Inflammatory Patterns

**DOI:** 10.3390/diagnostics10050282

**Published:** 2020-05-06

**Authors:** Jose A. Caparrós-Martín, Stephanie Flynn, F. Jerry Reen, David F. Woods, Patricia Agudelo-Romero, Sarath C. Ranganathan, Stephen M. Stick, Fergal O’Gara

**Affiliations:** 1 Human Microbiome Programme, School of Pharmacy and Biomedical Sciences, Curtin University, Perth 6102, Australia; jose.caparros-martin@curtin.edu.au; 2 Curtin Health Innovation Research Institute (CHIRI), Curtin University, Perth 6102, Australia; 3 BIOMERIT Research Centre, School of Microbiology, University College Cork, T12 YN60 Cork, Ireland; 110318793@umail.ucc.ie (S.F.); j.reen@ucc.ie (F.J.R.); david.woods@ucc.ie (D.F.W); 4 Telethon Kids Institute, The University of Western Australia, Perth 6009, Australia; Patricia.AgudeloRomero@telethonkids.org.au (P.A.-R.); Stephen.Stick@health.wa.gov.au (S.M.S.); 5 School of Biomedical Sciences, Faculty of Health and Medical Sciences, The University of Western Australia, Perth 6009, Australia; 6 Department of Respiratory Medicine, The Royal Children’s Hospital, Melbourne 3052, Australia; sarath.ranganathan@rch.org.au; 7 Infection and Immunity, Murdoch Children’s Research Institute, Melbourne 3052, Australia; 8 Department of Paediatrics, University of Melbourne, Melbourne 3010, Australia; 9 The Respiratory Research Centre, Telethon Institute for Child Health Research and School of Paediatrics and Child Health, The University of Western Australia, Perth 6009, Australia; 10 Department of Respiratory Medicine and Sleep Medicine, Perth Children’s Hospital, Perth 6009, Australia

**Keywords:** cystic fibrosis, bile acids, gut-lung axis

## Abstract

*Background*: Cystic fibrosis (CF) is a hereditary disorder in which persistent unresolved inflammation and recurrent airway infections play major roles in the initiation and progression of the disease. Little is known about triggering factors modulating the transition to chronic microbial infection and inflammation particularly in young children. Cystic fibrosis respiratory disease starts early in life, with the detection of inflammatory markers and infection evident even before respiratory symptoms arise. Thus, identifying factors that dysregulate immune responsiveness at the earliest stages of the disease will provide novel targets for early therapeutic intervention. *Methods*: We evaluated the clinical significance of bile acid detection in the bronchoalveolar lavage fluid of clinically stable preschool-aged children diagnosed with CF. *Results*: We applied an unbiased classification strategy to categorize these specimens based on bile acid profiles. We provide clear associations linking the presence of bile acids in the lungs with alterations in the expression of inflammatory markers. Using multiple regression analysis, we also demonstrate that clustering based on bile acid profiles is a meaningful predictor of the progression of structural lung disease. *Conclusions*: Altogether, our work has identified a clinically relevant host-derived factor that may participate in shaping early events in the aetiology of CF respiratory disease.

## 1. Introduction

Cystic fibrosis (CF) is an autosomal recessive condition caused by pathogenic genetic variants that compromise the function of the *cystic fibrosis conductance transmembrane regulator* (*CFTR*) gene product [1]. At the molecular level, the pathogenesis resulting from CFTR dysfunction involves the hyper-production of inspissated mucous secretions, which impact the integrity and function of the airway epithelial barrier [2,3]. In agreement with the widespread expression of *CFTR* [4], the clinical presentation of CF patients is linked to pleiotropic defects including pancreatic insufficiency, intestinal obstruction or liver and chronic airway disease.

Cystic fibrosis lung disease is characterized by chronic neutrophilic inflammation and infection, which progressively lead to the development of bronchiectasis, lung function decline and ultimately respiratory failure [5,6]. This progressive course is marked by a succession of unpredictable episodic clinical deteriorations known as acute pulmonary exacerbations, the aetiology of which is not well understood [7,8,9]. Therefore, delineating the factors predisposing CF patients to airway inflammation and infection in early life is a necessary prerequisite for improvements in the clinical management of respiratory lung disease particularly in children.

Apart from the evident respiratory phenotype, most chronic respiratory conditions develop parallel intestinal complications suggesting the existence of a gut-lung communication axis that can deliver aetiological factors into the lung epithelia [10,11]. These studies not only imply the existence of a bidirectional communication axis between both body sites through which alterations in one environment would be transmitted to the other, but also suggest common aetiological factors for both types of illnesses [10,11]. The most common gastrointestinal co-morbidity in chronic airway disease is duodenogastro-esophageal reflux, which has been associated with respiratory symptoms and disease progression in many respiratory conditions [12,13,14,15].

Among potential host triggers that may be implicated in modulating host-microbiota interactions and inflammation in the CF lung through the gut-lung axis, bile acids (BAs) have been demonstrated to influence both biofilm formation and antibiotic tolerance in a number of respiratory pathogens [16,17,18]. Bile acids have also been demonstrated to regulate the immune response of airway epithelial cells by repressing HIF1 signalling through destabilisation of HIF1-α and inducing the production of the pro-inflammatory cytokine interleukin 6 (IL6) [16,19]. We also observed that production of IL6 is regulated through the functional modulation of the BA receptor farnesoid X receptor (FXR) [16,19]. Bile acids are a key component of the gastric refluxate and are thought to gain entry into the CF lung through a (micro)aspiration process often associated with gastroesophageal reflux, which is a highly prevalent condition in CF patients [12,20,21,22].

The aim of this study was to evaluate whether the presence of BAs in the bronchoalveolar lavage fluid (BALF) of clinically stable preschool-aged children diagnosed with CF, was associated with markers of inflammation and clinical outcomes. In this work, we report a number of striking associations between the presence of BAs in the BALF of paediatric CF patients and the increase of proinflammatory cytokines. We also provide a statistical model validating BA detection in BALF as a significant predictor for accelerated progression of structural lung disease. Collectively these new data suggest that BAs could act to influence early events in the aetiology of CF respiratory disease. Progressing to dissect the CF lung-bile acid-inflammation axis will provide a mechanistic basis to address the pulmonary disease heterogeneity issue that is characteristic even in very early disease.

## 2. Material and Methods

### 2.1. BALF Collection, Cytology and Cytokine Measurements

BALF samples were collected according to the AREST-CF standard operating protocols [23]. Three washes of saline were lavaged into the right middle lobe (RML), and then one wash into the second most affected lobe determined by CT scan (usually lingula) [23]. Clinical microbiology was performed on the first RML and the left lobe washes [23]. The second and third RML lavage were pooled and processed into eukaryotic cells and supernatant. Viability of white cell lineages was determined using 0.1% trypan blue staining. Differential cell counts and cytokine measurements were previously described [23,24,25].

### 2.2. Bile Acid Profiling

BALF samples were treated with equal volumes of Sputolysin^®^ (Calbiochem, Darmstadt, Germany) and processed as described previously [16,26]. Each BALF sample was analysed blinded to patient data for the presence of 12 principal bile acids compared to purified reference standards. Cholic acid (CA), chenodeoxycholic acid (CDCA), deoxycholic acid (DCA), lithocholic acid (LCA), ursodeoxycholic acid (UDCA), glycodeoxycholic acid (GDCA), taurochenodeoxycholic acid, (TCDCA), taurodeoxycholic acid (TDCA), taurocholic acid (TCA), glycocholic acid (GCA), taurolithocholic acid (TLCA) were purchased from Sigma-Aldrich (Buchs, Switzerland) and tauroursodeoxycholic acid (TUDCA) was purchased from Calbiochem. All chemicals used were HPLC grade.

### 2.3. Statistical Analysis

Statistical analysis was carried out in R (version 3.4.2, R Foundation for Statistical Computing, Vienna, Austria) [27]. Hierarchical clustering was performed with the built-in function *hclust* using Ward’s criterion [28,29]. Silhouette information was obtained with the *silhouette* function of the package cluster [30]. Calinski-Harabasz index was calculated with the *NbClust* function of the package NbClust [31]. Principal component analysis was performed with the built-in function *prcomp*, which uses singular value decomposition for computation of the principal components. Contribution of each variable to the first principal component was calculated from the squared loadings for variables. Linear regression was performed using the built-in function *glm* using a Gaussian model and the *identity* link function. Linear mixed effect models were fitted using the *lme* function of the package lme4 [32]. Unless otherwise stated in the text and accordingly to widespread consensus, statistical tests’ null hypothesis was rejected when *p*-values were lower than 0.05.

### 2.4. Ethics, Consent and Permissions

Ethical approval (Ref. 1762/EPP) was previously granted to the AREST-CF program by the Princess Margaret Hospital for Children, Perth ethics committee (December 10, 2009) and written informed consent for publication was obtained to participate from parents/guardians. All methods performed in this study were carried out in accordance with the relevant guidelines and regulations

### 2.5. Data Availability

All relevant data are within the manuscript and its Supplementary Files.

## 3. Results

### 3.1. Clinical and Genetic Characterization of the Study Cohort

A cohort of 88 randomly selected bronchoalveolar lavage fluid (BALF) samples biobanked at the Australian Respiratory Surveillance Team for Cystic Fibrosis (AREST-CF) in Perth (WA, Australia) were used in this study. This paediatric cohort was from clinically stable preschool-aged patients ranging in age from 2.5 to 4.4 years (median age 3.25; interquartile range (IQR) 2.938–3.86). Most of the patients were diagnosed with CF at birth through newborn genetic screening (71 out of 88). The remaining subjects presented with a range of conditions including meconium ileus (nine), failure to thrive (seven), ecogenic fetal bowel (one), family history (one), or respiratory symptoms (two) including respiratory syncytial virus positive bronchiolitis (one). Subjects in this study were identified with the c.1521_1523delCTT (p.Phe508del) allele either in the homozygous (47%) or the heterozygous state. Those patients harbouring the p.Phe508del mutation in heterozygosity were described with a second pathogenic variant in the *CFTR* gene, with the exception of 4 cases in which a second deleterious allele was not reported (Supplementary Files). In cases in which the genetic screening was not positive CF diagnosis was confirmed by a chloride sweat test result greater than 60 mmol L^−1^.

Patients were examined annually at periods of stability in respiratory symptoms. The majority of samples were obtained during an annual follow up chest computed tomography (CT) scan procedure (86 out of 88), except for two of them that were collected following *Pseudomonas aeruginosa* eradication therapy. Clinical evaluation of the patients at the time of hospital visits included the collection of a BALF sample, as well as a routine CT scan procedure. The BALF specimens were collected from the right middle lobe following the established AREST-CF standard operating protocols. Cytokine and inflammatory markers testing, clinical biochemistry measurements, white blood cell count and viability, as well as clinical microbiology, were carried out on all of the BALF specimens immediately after collection following the standardized AREST-CF protocols [23,24,25]. BALF supernatants obtained after low speed centrifugation to pellet eukaryotic cells were then processed as single-use aliquots, snap frozen and stored until further analysis.

### 3.2. Classification of the Paediatric Cohort Based on Bile Acid Profiles in BALF

In order to investigate whether the presence of BAs in the lower airways of CF patients was associated with clinical outcomes, BALF samples were profiled for twelve individual BAs including conjugated primary and secondary BAs broadly representative of human bile [16,26,33]. The concentrations of BAs detected, although lower than those reported by our group and others in sputum or saliva [26,34], were consistent with previous reports from BALF [35] (Supplementary Files). Importantly, the total concentration of BAs was not correlated with either the volume of saline instilled into the lungs (Spearman *ρ* −0.08, *p*-value 0.43) or the volume of bronchial wash fluid recovered (Spearman *ρ* −0.11, *p*-value 0.29), suggesting that our data is truly representative of the BA load in the lower airways of these patients. 

**Figure 1:**
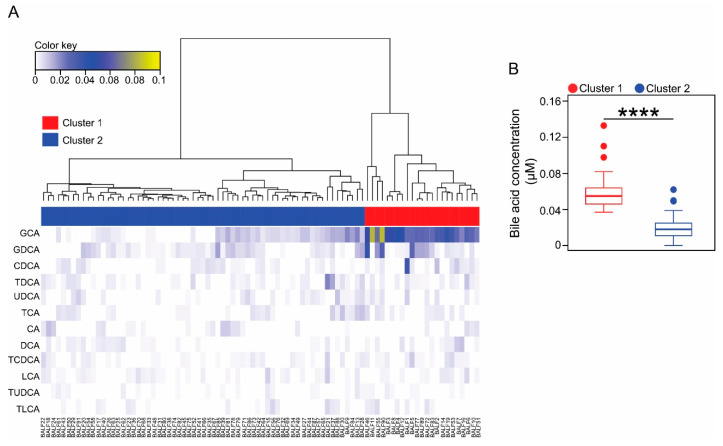
Cluster analysis of BALF samples based on their corresponding bile acids profiles. (**A**). Heatmap showing the bile acid profiles for each BALF sample included in this study. Columns represent individual samples and rows the different bile acid species profiled. An agglomerative hierarchical clustering approach was applied for grouping the samples based on Ward’s minimum variance method [28,29]. Colour key represents the concentration of each bile acid profiled in micromolar (µM) scale. The side colour bar indicates whether the sample was included in cluster 1 (red) or in cluster 2 (blue), after internal validation of the optimum number of clusters. (**B**). Boxplots show the total bile acid concentration for cluster 1 and cluster 2. Asterisks indicate statistical significance for the differences observed between clusters in the context of Mann-Whitney test. **** *p*-value < 0.0001.

To better understand the biological significance of BA detection in the BALF of CF patients, we grouped the samples into categories based on the resulting BAs profiles. For classification, we carried out an unsupervised clustering approach using an agglomerative hierarchical cluster model built applying Ward’s linkage method to a matrix of Euclidean distances. Thus, clustering is unbiased relying solely on the BAs profile of each of the samples. To evaluate the goodness of the different clustering solutions and therefore determine the optimal number of clusters from the model, we applied two clustering validity approaches; the Calinski-Harabasz index [36] and the silhouette coefficient [37,38]. A similar cluster validation approach was recently used for describing enterotypes in the human gut microbiota [39]. Both internal clustering validation measures, agreed that the 2-cluster outcome was the optimum solution for the sample classification problem (Figure 1A and Figure S1).

Next, we evaluated the characteristics of each cluster in the generated dendrogram. Upon closer inspection, we observed that BALF samples classified in cluster 1 were characterized by significantly higher levels of BAs than bronchial wash fluid specimens assigned to cluster 2 (cluster 1: Median 0.058 µM, IQR 0.046–0.067; cluster 2: Median 0.018 µM, IQR 0.011–0.025. Mann-Whitney test, *p*-value < 0.0001) (Figure 1B). Accordingly, the two clusters exhibited contrasting BA profiles. Thus, while the composition of cluster 1 was mainly dominated by glycocholic acid (GCA) and to a lesser extent by its conjugated dehydroxylated form glycodeoxycholic acid (GDCA), cluster 2 memberships demonstrated that these two BAs were less abundant (Figure S2). Samples classified in the two clusters were indistinguishable at the time of collection of the BALF specimen by age (Mann-Whitney test, *p*-value = 0.6) or the therapeutic regimen, including β-lactam antibiotic treatment (augmentin, χ^2^ 0.27, degrees of freedom (df) 1, *p*-value = 0.6 and cephalexin, χ^2^ 0.9, df 1, *p*-value = 0.34), non-antibiotic medications (omeprazole, χ^2^ 0.014, df 1, *p*-value = 0.9) or food supplements (calogen, χ^2^ 0.59, df 1, *p*-value = 0.44) among other prescribed medications (Supplementary Files).

This sample classification strategy provided us with a practical framework to evaluate the biological significance of bile acid detection in the lower airways of this paediatric CF cohort. Samples grouped in cluster 2 were used in this study as age- and disease-matched internal controls to assess significant relationships between bile acids and clinical outcomes. Following this comparative approach mitigated the necessity to consider involving non-CF samples in the analysis.

### 3.3. Cluster 1 Membership Is Associated with Inflammatory Markers

We next evaluated whether the partitioning of the BALF samples based on BA composition was associated with clinical outcomes in these patients. Samples in cluster 1 demonstrated significantly higher levels of interleukin 1 beta (IL1β) and IL6. In contrast no significant differences were observed in the production of neutrophil elastase (NE) or interleukin 8 (IL8) (Table 1). 

Total cell burden in BALF did not show any significant relationship between cluster membership and the relative abundance of specific immune cell lineages. Interestingly, we discovered that the viability of the white blood cells present in the bronchial wash fluid was significantly reduced in those specimens grouped in cluster 1 (Table 1). These observations are not confounded by intercluster differences in residual CFTR activity as indicated by the results of the sweat chloride test in these patients (cluster 1: median 90 mmol L^−1^, IQR 67.5–100.5; cluster 2: median 93 mmol L^−1^, IQR 67.5–102.25. Mann-Whitney test, *p*-value = 0.929) (Figure S3). Despite the presentation of a pro-inflammatory response, patients in cluster 1 did not exhibit a more severe lung structural deterioration as indicated by volumetric CT scan-based scores including the recently reported PRAGMA-CF metrics [40] (Table 2). 

Next, we investigated the detection of clinically relevant microorganisms that were cultured from the BALF specimens collected from each patient included in this study (Supplementary Files). According to the established AREST-CF protocols, clinical microbiology analysis is conducted separately for the first wash aliquot from the right middle lobe, and for the bronchial wash fluid from the second more affected lobe as per CT scan [23]. Data from both lobes was not combined but analysed separately. We considered the detection of 10,000 colony-forming units per millilitre as indicative of pathogen infection. This cut-off value has previously been shown to be a robust and reliable indicator of lower airway infection using BALF specimens [41]. Although we did not detect any significant association between infection by particular pathogens and cluster memberships in the right middle lobe, we did however observe that the samples in cluster 1 were significantly associated with greater odds for having positive cultures of oral flora in the left lobe (odds ratio OR 4.78, 95% confidence interval CI 1.62–14.15).

### 3.4. Cluster Membership Predicts the Progression of CF Lung Disease

Finally, we investigated whether cluster membership based on the detection of BAs in BALF could predict the progression of CF lung disease. For this purpose, we analysed the available clinical data on the patient cohorts obtained from the subsequent follow up medical evaluation visit using linear models. As expected, we observed that the proportion of the lungs with structural disease quantified using PRAGMA metrics [40], had significantly increased during this follow up year period in our patient cohort (Model 1, F(1,138) = 11.5, *p*-value < 0.05, multiple R^2^ 0.07) (Table S1). Introduction of the NE levels as an additional covariate resulted in a better fit of the model (Model 2 F(2,134) = 41.6, *p*-value < 0.05, multiple R^2^ 0.38), as indicated by the values of the Akaike Information Criterion (AIC) and maximum likelihood estimators (Table S1). This result supports earlier research showing that detection of NE activity in BALF of paediatric CF patients predicts the development and prognosis of bronchiectasis [6,42]. Interestingly, the likelihood of the previous model to predict the progression of lung disease was improved when the BA cluster membership was incorporated as an independent categorical variable, with all the coefficients of the linear model being statistically significant at α = 0.05 level. This new linear model predicts an increase in the proportion of the lung with structural disease over a period of one year, with a more pronounced progression in those patients belonging to cluster 1 (Model 3, F(3,133) = 31.1, *p*-value < 0.05, multiple R^2^ 0.41) (Table S1).

To account for interindividual variability in the response and therefore not to underestimate the total variance within our dataset, we also modelled the volume proportion of the lung with airway disease as a function of time using linear mixed models. The outcome of this analysis indicated that the fixed effects were approximately the same as for the standard linear regression. However, the likelihood of the resulting models using random effects for the intercept (Model 4, AIC 558), random effects for time (Model 5, AIC 558), or both (Model 6, AIC 562), was not improved with respect to the simplest model without random effects (Table S2).

The use of BA profiles as a predictor of the prognosis of CF lung disease requires further refinement for clinical consideration, particularly using longitudinal data. However, the predicted models indicate that the presence of bile acids in the lower airways of our patient cohort is associated with a less desirable disease trajectory in terms of lung structural damage.

## 4. Discussion

The present investigation provides experimental support to the hypothesis that BA (micro)aspiration could be an important factor in the pathophysiology and progression of CF lung disease in children. Bile acids are biological detergents that facilitate the digestion and absorption of lipids and fat-soluble vitamins [43]. Apart from these functions, they are also involved in many biological signalling and regulatory events, including metabolic tuning, modulation of the host immune responses, control of microbial communities, and regulation of pathogenicity mechanisms in respiratory pathogens [16,18,19,43,44,45,46,47]. Previous research has shown that the presence of BAs in BALF and sputum from CF patients and lung transplant recipients is associated with inflammatory markers, suggesting a role for BAs in the progression of CF respiratory disease and lung transplant rejection [34,35,48]. Our data expand these findings to paediatric CF patients where no consolidated structural lung disease was observed. Using an unbiased clustering approach based on BA profiles, we observed strong evidence associating the presence of BAs in BALF with inflammatory markers. We have reported that BAs elicit an immune response in epithelial cells with increased levels of cytokines in tandem with suppression of the HIF-1 signalling [16,19], which is known to be required for resolution of acute inflammation [49]. Previously, we have also demonstrated that BA-mediated induction of the pro-inflammatory cytokine IL6 was dependent on the BA receptor FXR [16]. This observation provides a plausible mechanism to the higher levels of IL6 observed in the BALF samples contained in cluster 1 of the present study. Furthermore, it has been shown in experimental animal models that sustained production of IL1β triggers neutrophil infiltration, NE production, and lung inflammation and fibrosis [50]. On the other hand, Esther and colleagues reported that the accumulation of inspissated mucus gels in CF airways relates to hypoxia and inflammation [51]. Since inhaled noxa spark the production of mucus by surface epithelial cells [52], bile acid translocation could exacerbate airway inflammation by stimulating mucus secretion. Altogether, these observations may help to explain the more pronounced progression of lung disease observed in those patients classified in the cluster with higher levels of BAs. Recently, new amino acid-conjugated cholic acid derivatives have been discovered and found to be enriched in CF patients [53]. These newly characterized BA species activate FXR and could further contribute to the dysregulation of the inflammatory response in CF [53]. Intriguingly, we also observed a reduced viability in the white blood cells recovered from cluster 1-associated BALF specimens. The homogeneity in the processing of BALF immediately after collection, and the unbiased techniques used for sample classification based on BAs profiles, make it very unlikely that this result could be as a consequence of the methodology employed. Bile acids are amphipathic molecules, some of which can disrupt the cellular membrane of eukaryotic cells, induce oxidative stress and promote apoptosis [54]. We have also demonstrated that bile activates a number of pathogenicity mechanisms including type-VI secretion systems in classic airway pathogens such as *Pseudomonas aeruginosa* [17]. Apart from being involved in interbacterial communication, in rare occasions type-VI secretion systems have also been shown to mediate host-pathogen interactions [55]. Moreover, the higher levels of IL1β in cluster 1 may underpin toll-like receptor- or nod-like receptor-mediated activation of caspase-1, which also triggers inflammatory programmed cell death [56]. Further studies are needed to ascertain whether the observed reduced viability is a consequence of direct toxicity or the modulation of selected pathogens in the lungs.

This work also raises a number of relevant questions regarding how BAs end up in the lungs of CF patients. On the basis of the well-established associations between reflux and lung function decline in many respiratory disorders, the available evidence would favour duodenal gastric reflux and (micro)aspiration as the major sources of BAs found in the BALF of this patient cohort [20,21]. The (micro)aspiration hypothesis is further supported by the fact that: *(i)* BAs can be detected in the gastric refluxate and saliva in humans [20,22], *(ii)* pepsin (another component of the gastric refluxate) is present in the BALF of CF individuals [57], *(iii)* compared to healthy people, occurrence of duodenal gastric reflux is high in CF patients [58] and a highly prevalent condition in CF children [21], and *(iv)* reflux and aspiration are primary to the development of respiratory symptoms and dictate the prognosis of lung disease [20]. In our cohort, BAs could also be derived from internal bleeding associated with the collection procedure, or the presence of structural lung disease. Bleeding caused by the medical procedure would be random. Thus, it would be very unlikely to observe the significant associations that we are reporting. The second option can also be discarded because in our cohort, patients discordant for BA concentration in BALF do have a similar degree of structural lung disease (Table 2). Furthermore, BAs in the systemic circulation mainly come from distal intestinal absorption, which is compromised in CF patients [59], and accordingly with newly synthesised BAs, we mainly detected the glycine-conjugated primary BA GCA (Figure 1 and Figure S2).

Overall, the present study shows that the presence of BAs in the lungs of paediatric CF patients associates with the modulation of a key event in the progression of lung disease, namely inflammation. Mucins and inflammatory markers in BALF are considered to be early drivers of structural lung disease in CF children [51]. It is currently not known if there is a relationship between BALF, mucus flakes and bile acid profiles, and future mechanistic approaches will address this possibility. The outcome of such studies could have important implications for developing possible preventative treatments to delay the course of CF disease. The cross-sectional design of this study and the lack of a non-CF control cohort do not permit us to establish the nature of the causal links or to determine whether the translocation of BAs into the lungs is exclusive of children with CF. While we acknowledge this limitation, the reported association results support the need for further investigations involving longitudinal and animal model studies to dissect the mechanisms through which BAs modulate the progression of CF lung disease.

## Figures and Tables

**Table 1:** Quantification of inflammatory markers and white cell composition in the BALF samples analysed in this study. Data represents the median value and the interquartile range (1st quartile-3rd quartile) of each variable per cluster. Mann-Whitney test was used for pairwise comparisons between group levels. Family-wise error rate was controlled using Bonferroni-corrected *p*-values.

Variable	Cluster 1	Cluster 2	*p*-Value
**NE (ng mL^−1^)**	100 (100–100)	100 (100–100)	1
**IL8 (pg mL^−1^)**	1075 (300–2150)	810 (250–1890)	1
**IL1β (ng mL^−1^)**	83.8 (54.44–122.52)	10 (10–32.58)	0.006
**IL6 (ng mL^−1^)**	57.79 (34.73–77.65)	10 (10–10)	0.041
**Total cell count**			
**(x10^6^ cells mL^−1^ BALF)**	0.32 (0.23–0.57)	0.29 (0.18–0.38)	1
**% viability**	74.8 (62.4–80.05)	82.8 (73.1–87.3)	0.038
**% macrophages**	83.33 (61.58–93.42)	86.67 (70.33–94)	1
**% neutrophils**	16.33 (6.58–38.24)	12 (4.33–29)	1
**% lymphocytes**	0 (0–0.33)	0 (0–1.67)	1
**% eosinophils**	0 (0–0.33)	0 (0–0.3)	1

**Table 2:** Quantification of structural lung disease at the time of the collection of the BALF samples from patients classified in cluster 1 or cluster 2. Lung disease was scored using either volumetric CT scans or the recently reported PRAGMA-CF methodology [40]. Calculated probabilities for rejecting the null hypothesis of samples coming from the same population in the context of the Mann-Whitney test were corrected for multiple comparisons using Bonferroni approach. BW, bronchial wall; Mu, Mucus; %Dis, volume proportion of the lung with airway disease [40]; % Bx, volume proportion of the lung with bronchiectasis [40]; %TA, volume proportion of the lung with trapped air [40], % Atelec, volume proportion of the lung with atelectasis [40].

Variable	Cluster 1	Cluster 2	*p*-Value
**Bronchiectasis score**	1 (0–2)	1 (0–3.5)	1
**BW thickening score**	10 (7–10)	8 (5–10)	1
**Air trapped score**	1 (0.75–4)	2 (1–4)	1
**Mu. plugging score**	0 (0–0.25)	0 (0–1)	1
**% Dis**	2.99 (2.39–4.13)	2.17 (1.1–3.8)	0.94
**% Bx**	0 (0–0.075)	0 (0–0.38)	1
**% TA**	2.06 (0.96–6.14)	1.28 (0.19–5.86)	1
**% Atelec**	0.06 (0–1.77)	0.2 (0–0.97)	1

## Supplementary Materials

The following are available online at https://www.mdpi.com/2075-4418/10/5/282/s1, Supplementary Files: Sample metadata and bile acid profiles in BALF. Figure S1: Average silhouette coefficient for the indicated number of clusters. Figure S2: Bile acid composition in the two groups of BALF samples. Figure S3: Sweat chloride concentration for patients assigned to each cluster. Table S1: Regression results for the linear models. Table S2: Regression results for the linear mixed effect models.
