# Supplementary material for: The Detection of Bile Acids in the Lungs of Paediatric Cystic Fibrosis Patients Is Associated with Altered Inflammatory Patterns
Source: Diagnostics (Basel). 2020 May 6;10(5):282. doi: 10.3390/diagnostics10050282 (PMC7277992; doi:10.3390/diagnostics10050282)
Supplement: Supplementary file 1 [file diagnostics-10-00282-s001.zip › diagnostics-760450-supplementary/Caparros-Martin_SuppMaterial.pdf]

SUPPLEMENTAL MATERIAL

THE DETECTION OF BILE ACIDS IN THE LUNGS OF PAEDIATRIC CYSTIC FIBROSIS PATIENTS IS ASSOCIATED WITH ALTERED INFLAMMATORY PATTERNS

Jose A. Caparrós-Martín, Stephanie Flynn, F. Jerry Reen, David F. Woods, Patricia Agudelo-Romero, Sarath C. Ranganathan, Stephen M. Stick and Fergal O’Gara.

Supplemental Figure 1

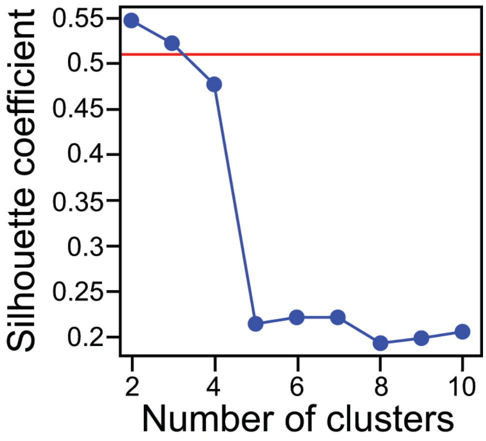

Supplemental figure 1. Average silhouette coefficient for the indicated number of clusters. Line chart showing the quality of clustering achieved for the number of clusters indicated in the X-axis. The horizontal red line represents the cut-off value below which the overall structure of the clustering solution is considered weak [1, 2]. Accordingly to this interpretation of the average silhouette coefficients, a reasonable structure is observed for 2 and 3 cluster solutions [1, 2].

Supplemental Figure 2

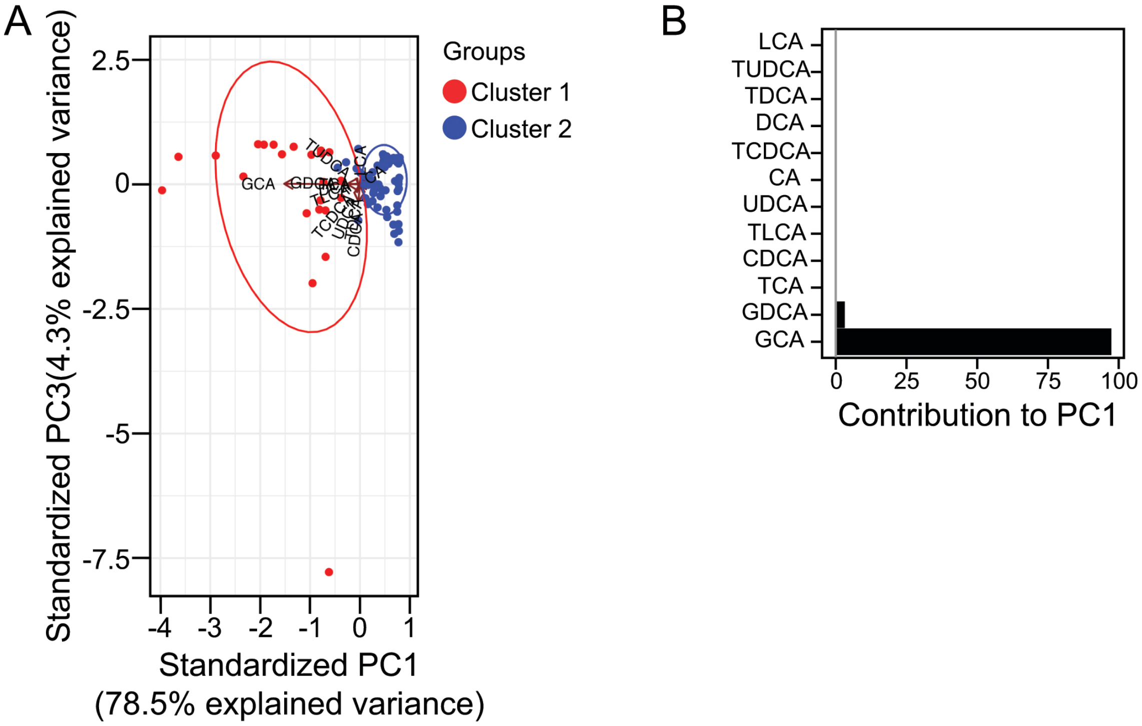

Supplemental figure 2. Bile acid composition in the two groups of BALF samples. A. Principal component analysis in the bile acid profiles of the BALF samples. Two components (PC1 and PC3) were extracted accounting for over 80% of the variance in this dataset, with PC1 providing a good separation between the samples assigned to the two clusters. Dots represent each sample in cluster 1 (red) or cluster 2 (blue). Ellipses delimit 68% confidence interval. **B.** Proportion of variance of each bile acid species explained by PC1 was calculated using the squared loadings.

Supplemental Figure 3

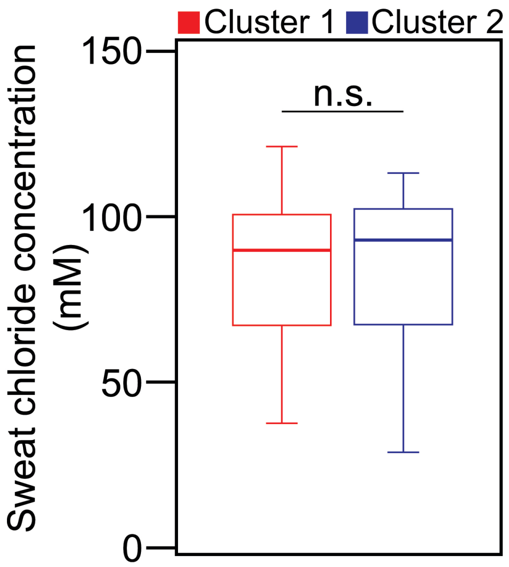

Supplemental figure 3. Sweat chloride concentration for patients assigned to each cluster. Boxplot represents the sweat chloride test results for the patients included in this study. Differences between clusters were assessed for statistical significance using the Mann-Whitney test. n.s., not significant.

Supplemental table 1.

	Proportion of lung with structural disease		
	**Model 1**	**Model 2**	**Model 3**
Time	1.2^{***} (0.51, 1.9)	1.1^{***} (0.57, 1.7)	1.2^{***} (0.6, 1.7)
Neutrophil elastase (ng/ml)		0.001^{***} (0.001, 0.001)	0.001^{***} (0.001, 0.001)
Cluster membership (cluster 2 = 1)			-0.87^{*} (-1.5, -0.21)
Intercept	1.6^{***} (0.51, 2.7)	1.2^{***} (0.34, 2.2)	1.9^{***} (0.85, 2.9)
Number of observations	140	137	137
Log Likelihood	-303	-268	-265
AIC	609	542	538

Supplemental table 1. Regression results for the linear models. Regression coefficients are provided as value (95% confidence interval). Since cluster membership is a binary predictor, this variable takes value 1 when the sample belongs to cluster 2, and 0 when it is grouped in cluster 1. Asterisks indicate the conditioned probability for a zero correlation between the response and the independent variables. $^{***}P < 0.001$; $^{*}P < 0.05$.

Supplemental table 2.

	Proportion of lung with structural disease		
	**Model 4**	**Model 5**	**Model 6**
Time (years)	1.2^{***} (0.65, 1.7)	1.2^{***} (0.64, 1.7)	1.2^{***} (0.65, 1.7)
Neutrophil elastase (ng/ml)	0.001^{***} (0.001, 0.001)	0.001^{***} (0.001, 0.001)	0.001^{***} (0.001, 0.001)
Cluster membership (cluster 2 = 1)	-0.86^{*} (-1.6, -0.15)	-0.85^{*} (-1.5, -0.16)	-0.85^{*} (-1.6, -0.15)
Intercept	1.9^{***} (0.89, 2.8)	1.9^{***} (0.9, 2.8)	1.9^{***} (0.9, 2.8)
N	137	137	137
Log Likelihood	-273	-273	-273
AIC	558	558	562

Supplemental table 2. Regression results for the linear mixed effect models. Regression coefficients are provided as value (95% confidence interval). Since cluster membership is a binary predictor, this variable takes value 1 when the sample belongs to cluster 2, and 0 when it is grouped in cluster 1. Asterisks indicate the conditioned probability for no correlation between the response and the independent variables. $^{***}P < 0.001$; $^{*}P < 0.05$.

Supplemental references

1. Kaufman L, Rousseeuw PJ: **Partitioning around medoids (program PAM).** In: *Finding groups in data, an introduction to cluster analysis.* John Wiley and Sons, Hoboken NJ 1990; 1990: 83-88.
2. Rousseeuw PJ: **Silhouettes - a Graphical Aid to the Interpretation and Validation of Cluster-Analysis.** *J Comput Appl Math* 1987, 20:53-65.
